# Coumarins: Quorum Sensing and Biofilm Formation Inhibition

**DOI:** 10.3390/molecules29194534

**Published:** 2024-09-24

**Authors:** Eslam R. El-Sawy, Mohamed S. Abdel-Aziz, Heba Abdelmegeed, Gilbert Kirsch

**Affiliations:** 1 Chemistry of Natural Compounds Department, Pharmaceutical and Drug Industries Research Institute, National Research Centre, Giza 12622, Egypt; hn.abdelmegeed@nrc.sci.eg; 2 Microbial Chemistry Department, Biotechnology Research Institute, National Research Centre, Giza 12622, Egypt; mohabomerna@yahoo.ca; 3 Laboratoire Lorrain de Chimie Moléculaire (L.2.C.M.), Université de Lorraine, 57050 Metz, France

**Keywords:** coumarins, quorum sensing, biofilm formation inhibition, natural coumarins, synergistic effect

## Abstract

Quorum sensing (QS) is a bacterial cell-to-cell communication mechanism that plays an essential role in bacterial pathogenesis. QS governs bacterial behavior and controls biofilm formation, which in turn contributes to antibiotic resistance. Therefore, identifying and synthesizing novel compounds to overcome QS and inhibit biofilm formation are essential. Coumarins are important plant-derived natural products with wide-ranging bioactivities and extensive applications, including antibacterial, antifungal, anticoagulant, antioxidant, anticancer, and anti-inflammatory properties. Additionally, coumarins are capable of QS rewiring and biofilm formation inhibition, leading to higher susceptibility to antimicrobial agents and less antibiotic resistance. Therefore, in this review, we aim to provide an overview of QS and biofilm formation. This review also discusses the role of natural and synthesized coumarins in controlling QS, inhibiting biofilm formation, and inducing synergy in antibiotic–coumarin combinations. Hence, this review emphasizes the potential of coumarin compounds to act as antibacterial agents and demonstrates their ability to alleviate antibiotic resistance.

## 1. Introduction

Bacterial adaptability to the complicated, constantly changing host environment is crucial for their pathogenesis. Therefore, bacteria have the ability to recognize and respond to these environmental changes by producing different signals. Such bacterial signals facilitate cellular communication within a single population of bacteria (interspecies communication) or between different bacterial populations (interspecies communication). Additionally, they enable bacteria to modify the expression of specific genes and rewire their metabolic pathways to accommodate their needs [1]. Quorum sensing (QS) is a major molecular mechanism that is involved in bacterial adaptation to the host environment [2,3]. QS is bacterial cell-to-cell communication that plays a critical role in microbial communities. Microbial communication through QS occurs via many chemical signals called autoinducers [4,5]. Autoinducers are small molecules produced by bacteria that are responsible for transferring different information and signals across the microbial community. Therefore, they control bacterial density, competence, virulence, resistance to antibiotics, and biofilm formation [3,4,5,6,7]. Autoinducers are divided into three main categories; acyl homoserine lactones (AHLs), which primarily exist in Gram-negative bacteria; autoinducer oligopeptides, which are predominant in Gram-positive bacteria’ and autoinducer-2 (AI-2), a furanosyl borate diester, that was first discovered in the Gram-negative, bioluminescent marine bacterium *Vibrio harveyi* [8]. Through these autoinducers, bacteria can regulate their behavior and cellular pathways according to their population density and in response to changes in the host environment. 

One of the main processes controlled by QS is biofilm formation. Bacterial biofilm is a mode of growth where bacteria are frequently found encased in a polysaccharide matrix attached to a solid surface. Hence, biofilm formation offers protection from environmental agents and ensures bacterial survival [9]. It has been shown that bacterial cells that inhabit biofilms exhibit 10 to 1000 times less susceptibility to antimicrobial agents [10]. This poor susceptibility is attributed to poor antibiotic permeability due to less penetration of the bacterial biofilm. The high population densities and proximity of cells in biofilms also increase the chances for genetic exchange of antimicrobial resistance genes [11]. Additionally, biofilm is considered a special microenvironment that harbors heterogeneous areas with different concentrations of nutrients and bacterial secretions. Such heterogeneity leads to different levels of bacterial resistance [3,7]. Thus, biofilm formation is a central mechanism that confers antibiotic resistance, which we need to overcome to improve bacterial susceptibility to antimicrobial agents.

Over the past decades, there has been increasing interest in the potential human health benefits of natural compounds. Therefore, identifying and developing novel compounds from natural sources represent a safer approach for combating microbial resistance and inhibiting biofilm formation due to their fewer side effects. 

The most promising anti-QS molecules and inhibitors of biofilm formation found in plants are from the coumarin family, a large structurally diverse family of plant phenolic compounds characterized by various pharmacological properties.

**Figure 1:**
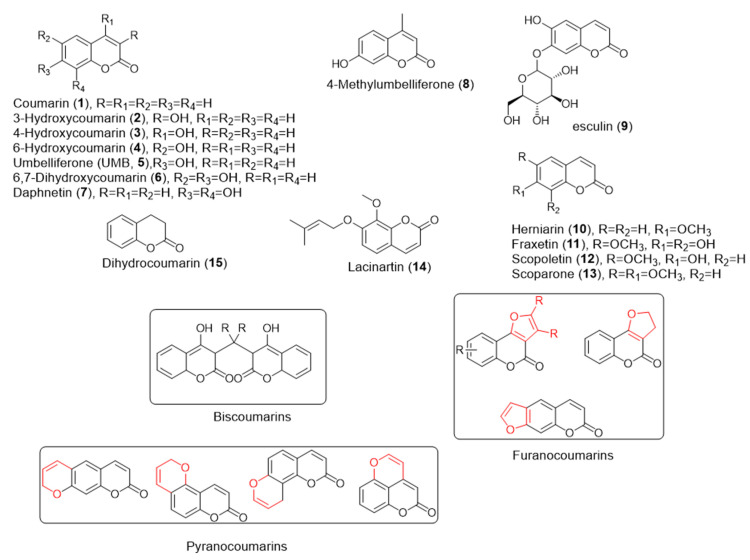
The chemical structures of some simple and complex coumarins.

Coumarin is a natural compound that was isolated from *Coumarouna odorata* in 1820 by Vogel. It belongs to the group of simple phenolic compounds found in plants, microorganisms, and some animals [12]. Some plant families like Umbelliferae, Rutaceae, Compositae, Leguminosae, Oleaceae, Moraceae, and Thymelaeaceae produce high levels of coumarin compounds. 2H-1-benzopyran-2-one is the basic structure of coumarin (**1**), and coumarins are further classified into simple and complex coumarins based on this structure. Simple coumarins include 3-hydroxy (**2**), 4-hydroxy (**3**), 6-hydroxy (**4**), 7-hydroxy (umbelliferone, **5**), 6,7-dihydroxycoumarins (**6**), daphnetin (**7**), 4-methylumbelliferone (**8**), esculin (**9**), herniarin (**10**), fraxetin (**11**), scopoletin (**12**), scoparone (**13**), and lacinartin (**14**). Complex coumarins include biscoumarins, furanocoumarins, pyranocoumarins, and dihydrofurocoumarins [12,13,14,15] (Figure 1).

Coumarin and its derivatives have diverse biological activities, including anti-tumor [16], anti-inflammatory [17], anticoagulant [18], antiviral [19,20], antimicrobial [21,22], and insecticidal activity [23]. One of the most important biological features that distinguish the coumarin family is that they have been identified as potent anti-virulent compounds [24] and as inhibitors of biofilm formation of a wide range of pathogens. This includes ESKAPE pathogens such as *Staphylococcus epidermidis*, *Porphyromonas gingivalis*, *Escherichia coli*, *Salmonella typhimurium*, *Candida albicans*, and *Pseudomonas aeruginosa* and the phytopathogen *Ralstonia solanacearum* [25,26].

Through our current review, we will shed light on QS, explain the mechanisms of biofilm formation, and demonstrate the relationship between QS and biofilm formation. Moreover, we will describe the importance of natural and synthesized coumarins that combat QS and biofilm formation, considering the literature was published from 2011 until now.

## 2. Quorum Sensing (QS)

QS is a route enabling bacterial cells to communicate chemically that relies on the production, detection, and response to extracellular signaling molecules known as autoinducers. QS permits groups of bacteria to alter their behaviors in response to changes in the population density and species composition of the vicinal community [27]. QS, as a communication mechanism, is found in all kinds of microorganisms [28]. It regulates gene expression in response to variations in cell population density. Through QS, both Gram-positive and Gram-negative bacteria form communication circuits to control different ranges of bacterial characteristics, such as virulence, conjugation, competence, antibiotic production, sporulation, motility, and biofilm formation [29]. Bacteria produce and release small molecules as chemical QS signals known as autoinducers. Autoinducers of Gram-negative bacteria are acylated homoserine lactones (AHLs), whereas Gram-positive bacteria use oligopeptides to communicate [3]. Recent investigations in this field indicated that cell–cell communication through autoinducers occurs either within the same bacterial species or between different bacterial species [30]. Additionally, there are increasing investigations suggesting that bacterial autoinducers provoke exact responses in their hosts. It has been found that the bacterial ability to communicate with each other allows them to coordinate gene expression, thus modulating the bacterial behavior of the entire community [27,31]. Nearly 80% of microbial infections are related to biofilm formation, which is controlled by QS [32]. It has been shown that, compared to free single cells, bacteria that are found in biofilms are more resistant to antibiotics, environmental pressure, and the host immune system [33].

### 2.1. Quorum Sensing in Gram-Negative Bacteria

**Figure 2:**
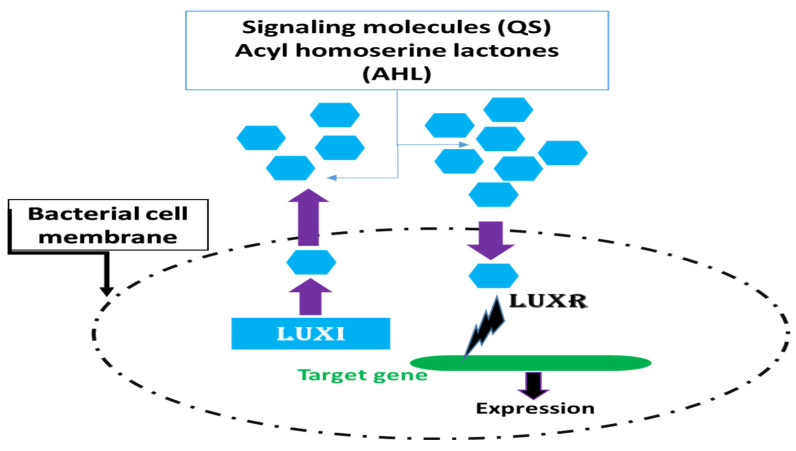
Quorum sensing in Gram-positive bacteria.

AHLs are the QS signaling molecules found in Gram-negative bacteria, with autoinducer 1 (AI-1) serving as an example. They are water-soluble molecules that can easily permeate through bacterial cellular membranes. Consequently, AHLs freely enter and leave the bacterial cells to maintain constant concentrations inside and outside [34,35]. The LuxR–LuxI system is the typical regulatory system of Gram-negative bacteria. A monomeric LuxI homolog catalyzes the synthesis of an AHL. When the AHL reaches a critical concentration, it can bind to a LuxR homolog and stabilize the active dimeric protein. The AHL-bound LuxR can bind to QS-activated promoters of specific DNA target sites to adjust the expression of the target genes [36] (Figure 2).

### 2.2. Quorum Sensing in Gram-Positive Bacteria

**Figure 3:**
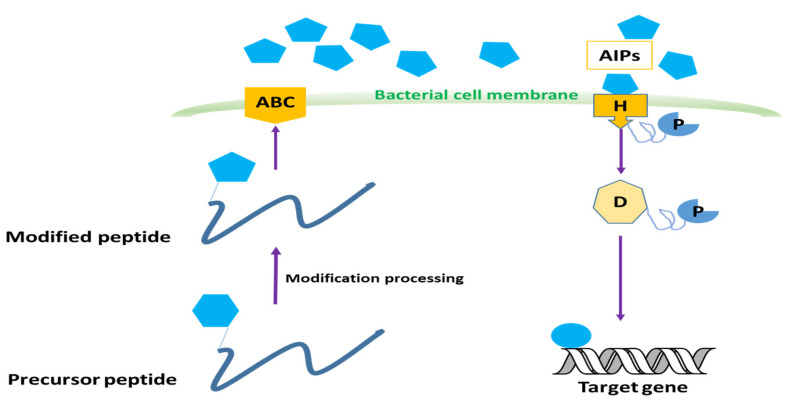
Quorum sensing in Gram-negative bacteria.

Autoinducer peptides (AIPs) are the signaling molecules of Gram-positive bacteria that are recognized by histidine kinases (receptors) on the membrane or in the cytoplasm. The QS system in Gram-positive bacteria is divided into two parts. The first part is a specific autoinducer peptide transport system, and the second part is a regulatory signal transduction system [37,38]. Through a series of changes and processes, the peptide precursor becomes a stable active signaling molecule (AIP) that is secreted into the extracellular environment with the help of enzyme ABC transferase (ATP-binding cassette transporter). When the extracellular secretion of AIPs reaches the needed concentration, it interacts with the phosphokinase biocomponent on the cell membrane. This interaction between phosphokinase and AIP leads to the autophosphorylation of retained histidine residues (H) followed by the phosphorylation of the aspartic acid residues (D) in the regulatory protein that responds to it. Lastly, the phosphorylated response regulatory protein interacts with specific DNA target sites to adjust the expression of the target genes [39] (Figure 3).

### 2.3. Detection of Quorum Sensing Signaling Molecules

QS signal molecules can be detected using three different methods. First, *Chromobacterium violaceum*, *Agrobacterium tumefaciens*, *E. coli,* or *Vibrio fischeri* biosensors are used to sense N-acyl homoserine lactones (AHLs) [40]. Bacterial biosensors can be induced by strains containing AHLs to produce distinguishable phenotypic changes, including large-scale expression of purple color and/or β-galactosidase. Second, QS can be detected using chromatographic techniques such as thin layer chromatography (TLC) and high-performance liquid chromatography (HPLC) [41]. Third, a combination of the TLC method with a bacterial biosensor (TLC-Biosensor) can also be used to detect QS signal molecules [42].

### 2.4. Qs as a Chemical Tool in Enhancing Biofilm Formation in Bacteria

**Figure 4:**
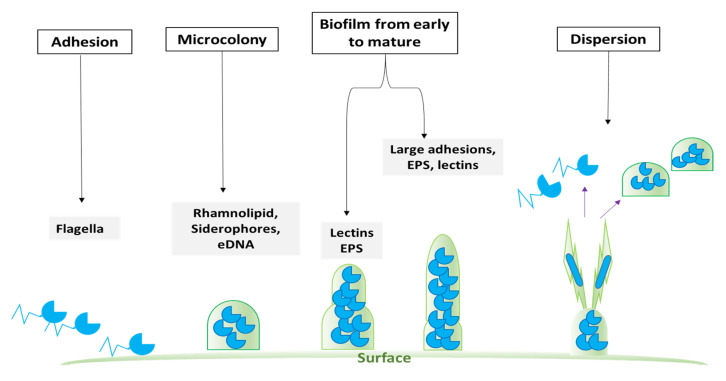
Stages of biofilm formation from initiation to dispersion.

The colony behavior of bacterial cells is controlled by both QS and biofilm formation. The signal transmission of autoinducers between cells leading to the detection of bacterial cell density is known as QS, while biofilm is known as bacterial cell aggregation. The cell aggregates are quite similar and connected to each other through bacterial cell self-excretion of an extracellular matrix known as biofilm; thus, biofilm is known as an environment controlled by QS [43]. *Streptococcus oralis* 34 secretes autoinducer (AI-2), which is necessary for biofilm formation by *S. oralis* 34 and *Actinomyces naeslundii* [44]. AI-2 was also found to be a mediator for QS in *E. coli*; thus, it is involved in biofilm formation, regulation of motility genes, synthesis of flagellum, and chemotaxis [45]. Biofilm formation in bacteria is an active and layered process. The first and crucial stage of the formation of bacterial biofilm is adhesion to the host, which is followed by the biofilm aggregation stage, maturation, and dispersion (Figure 4). Bacterial cells adhere mainly by binding to the host surface through the outer membrane adhesive proteins and by excreting exopolysaccharides (EPSs) [46]. Furthermore, QS is involved in the dynamic process of biofilm formation in both Gram-negative and Gram-positive bacteria [47]. QS in *P. aeruginosa* (as a representative Gram-negative bacteria) is complicated and is judged by transcriptional regulators MvaT and RsaL. It is also controlled by post transcriptional regulators (RsmA), sigma factors (RpoN and RpoS), and 2-heptyl-3-hydroxy-4(1H)-quinolone (PQS) systems [43]. In addition to the functions that are controlled by LasI/LasR/RhII/RhIR, there are several virulence factors responsible for biofilm formation, such as rhamnolipid, lectin, and siderophores [48]. Surface movement (clustering) is maintained by rhamnolipid, leading to biofilm formation. Additionally, LecA and LecB are considered QS-dependent carbohydrates (bending lectins) that exhibit an essential role in biofilm formation in mutants that cannot produce biofilm [49,50]. The PQS activates the production of extracellular DNA (eDNA) that interacts with exopolysaccharides (positively charged molecules) in the matrix to form the biofilm [51]. 

Biofilm is also known to be produced by *Staphylococcus aureus* (a Gram-positive representative) through the AIP-mediated QS sensing system. Additionally, the Agr QS system, where the *agr* locus encodes a QS circuit, inhibits the formation of biofilm; therefore, the biofilm formed by the agr-carrying strain of *S. aureus* is thinner than that by the agr-deficient strain. The Agr system upregulates the expression of δ-hemolysin (cleaved mature biofilms) and downregulates the expression of adhesion proteins that play an important role in maintaining the integrity of biofilms [52]. Furthermore, RNAIII inhibiting peptide (RIP, YSPWTNF) is a heptapeptide that interferes with the biofilm formation of *S. aureus* by preventing the agr system [53]. In the LuxS/AI-2 QS system of *S. aureus*, the deletion of the LuxS gene could lead to enhanced biofilm inhibition and antibiotic sensitivity, thus affecting the pathogenicity of the strain [54]. This biofilm inhibition may be due to the ability of the luxS gene to downregulate the genes related to biofilm formation. Moreover, environmental conditions such as carbon sources, pH, and flow rates regulate QS and affect biofilm formation as well [27]. It was shown that AHLs of Gram-negative bacteria are fairly stable at acidic and neutral pH. In contrast, at high pH, chemical hydrolysis of the homoserine lactone ring occurs. Regardless of whether the QS regulating biofilm formation is negative or positive, the bacterial biomass required to initiate QS in a given bacterial population increases with the fluid environment flow rate [55]. 

## 3. QS Activity and Biofilm Formation Inhibition of Natural Coumarins

Coumarins are a family of phenolic compounds that are derived from plant sources. They have received considerable attention due to their antimicrobial properties. Thus, coumarins are emerging as promising candidates for developing next-generation antimicrobial agents. One of the most important biological features of the coumarin family is that they act as potent antivirulent compounds [24] and inhibit biofilm formation in a wide range of pathogens [25,26]. Table 1 presents natural coumarin derivatives that have shown QS activity and inhibit biofilm formation.

The anti-QS activity of coumarin (**1**) is mainly due to its effect on various Gram-negative bacteria that utilize type 1 density-dependent communication systems [25]. This includes its impact on *P. aeruginosa* by inhibiting biofilm formation, phenazine biosynthesis, and motility. On the other hand, the effect of coumarin (**1**) on *Aliivibrio fischeri* involves inhibition of bioluminescence, which is caused by transcription of the lux operon (which is induced through population-dependent quorum sensing) [25]. It is important to note that the reduction in biofilm formation by coumarin (**1**), unlike antibiotics that aim to inhibit cell growth, is due to its antibiofilm activity and not its antimicrobial activity [72]. Hence, certain coumarins that act as biofilm inhibitors but do not inhibit bacterial growth could reduce the risk of drug resistance [72].

Another simple coumarin, dihydrocoumarin (**15**), exhibited activity that effectively suppressed the biosynthesis of quorum-dependent violacein, a blue-violet pigment, in *C. violaceum* and biofilm formation in *Hafnia alvei* [71]. 

**Figure 5:**
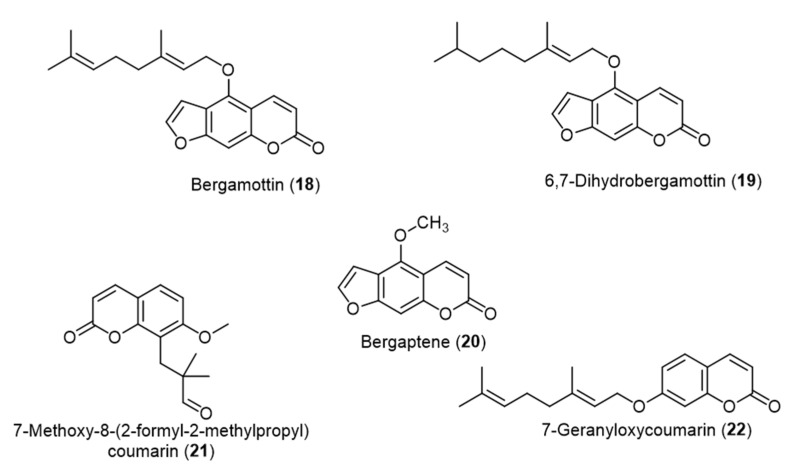
The chemical structures of coumarins isolated from citrus fruits.

In a distinctive study, the effect of some members of the coumarin family (coumarin (**1**), 4-hydroxycoumarin (**3**), umbelliferone (**5**), 6,7-dihydroxycoumarin (**6**), daphnetin (**7**), and scopoletin (**13**)) on biofilm formation in *E. coli* O157:H7 was investigated. The study confirmed the antibiofilm effect of the identified coumarins and demonstrated for the first time the distinct antibiofilm properties of umbelliferone (**5**) [56]. In another study, two furocoumarins, bergamottin (**18**) and 6,7-dihydrobergamottin (**19**) isolated from grapefruit juice as well as citrus fruits (*Citrus bergamia, Citrus maxima, and Citrus paradisi*), suppressed QS biofilm formation in *E. coli O157:H7* by 72 and 58.3%, respectively (Figure 5). The two furocoumarins (18 and 19) exhibited their activity vis-à-vis bacteria that use both AI-1 (N-acyl homoserine lactones, AHLs) and AI-2 (furanosyl borate diester) signaling through density-dependent communication [73]. 

While the furocoumarins 7-methoxy-8-(2-formyl-2-methylpropyl) coumarin (**20**), bergaptene (**21**), and 7-geranyloxycoumarin (**22**) (Figure 5), along with flavonoids from grapefruit (*Citrus paradisi)* essential oils, did not inhibit the growth of *P. aeruginosa*, they did inhibit biofilm formation by 52% and 55%, sessile cell viability by 45% and 48%, autoinducer production, and elastase activity by 30% and 56% [74]. 

In 2017, D’Almeida and his colleagues studied the inhibitory effects of coumarin (**1**) and six structurally related compounds: 3-hydroxy (**2**), 4-hydroxy (**3**), 6-hydroxy (**4**), 7-hydroxy (**5**), 6,7-dihydroxy coumarins (**6**), and dihydrocoumarin (**15**) on the QS of *P. aeruginosa* and *C. violaceum*. They demonstrated that the coumarins with hydroxyl groups on the phenyl ring displayed higher activity against *P. aeruginosa* biofilm formation than the coumarins with hydroxyl groups on the pyrane ring or dihydrocoumarin. In addition, 3-hydroxy and 4-hydroxy coumarins showed a decrease in their antibiofilm activity compared with coumarin itself. Additionally, 3-hydroxycoumarin was important for the inhibition of *P. aeruginosa* and *C. violaceum* QS. These observed effects were active independently of any effect on growth, indicating that hydroxycoumarins are useful as anti-virulence agents against *P. aeruginosa* and *C. violaceum* [75].

Coumarin (**1**) and 6,7-dihydroxycoumarin (**6**) were identified as components of *Acacia* species collected from Benin and obtained using the ethanol extraction process. These compounds showed antimicrobial activity with MIC values ranging from 0.1562 mg/mL to 2.5 mg/mL against *S. aureus*, *Enterococcus faecalis*, *P. aeruginosa*, *Salmonella typhi*, and *C. albicans,* with excellent biofilm inhibition at MIC and sub-MIC concentrations, especially against *E. coli* [76]. Additionally, the extracts were able to disrupt QS processes in *C. violaceum* CV12472 and *C. violaceum* CV026 as they inhibited violacein production, possibly through modulating signal production and reception mechanisms [76].

## 4. QS Activity and Biofilm Formation Inhibition of Synthetic Coumarin Compounds 

The inhibition of QS and the inhibition of biofilm formation by coumarin and its derivatives have encouraged researchers to develop the coumarin ring by constructing new coumarin compounds, searching for derivatives that counter or reduce the risk of bacterial resistance, and then determining their mechanism of action as biofilm inhibitors.

**Scheme 1:**
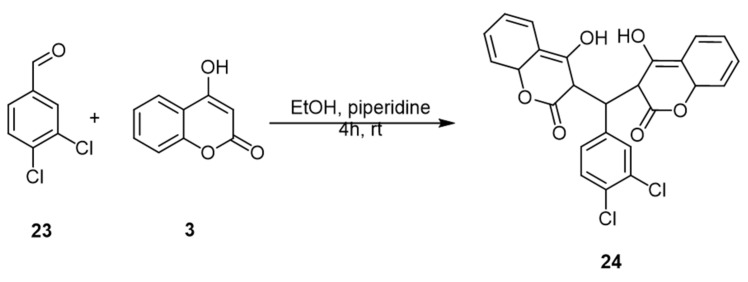
Synthesis of a biscoumarin derivative (DCH, **24**).

In unique research, Qu et al. prepared a series of bis-coumarin derivatives [77] via the reaction of substituted benzaldehyde and 4-hydroxycoumarin (**3**) in the presence of a catalytic amount of piperidine (Scheme 1). One biscoumarin derivative (DCH, 24) showed unique activity against a broad range of Gram-positive and Gram-negative bacteria, as well as methicillin-susceptible *S. aureus* (MSSA) and methicillin-resistant *S. aureus* (MRSA) strains. The results indicated that DCH (24) showed antimicrobial activity against Gram-positive bacteria such as *S. aureus* and *S. epidermidis* with MIC values ranging from 4 to 32 μg/mL and was ineffective against Gram-negative bacterial strains [77]. Additionally, DCH (**24**) inhibited MRSA with a MIC of 4 or 8 μg/mL in comparison to a quinolone antibacterial agent, which significantly inhibited the growth of *S. aureus* ATCC 29213 at 8 μg/mL [77]. Next, they studied the mechanistic pathway of its action against MRSA and indicated that DCH (**24**) at a concentration range of 0.25 to 4 μg/mL (below the MIC) inhibited MRSA biofilm by inhibiting the expression levels of genes including *srtA*, *atlE*, *aap*, and *icaA* [77]. In addition, DCH (**24**) inhibited MRSA adhesion and biofilm formation on the catheter surface and inhibited the spread and growth of MRSA from the catheter to the liver, spleen, lung, and kidney [77].

**Scheme 2:**
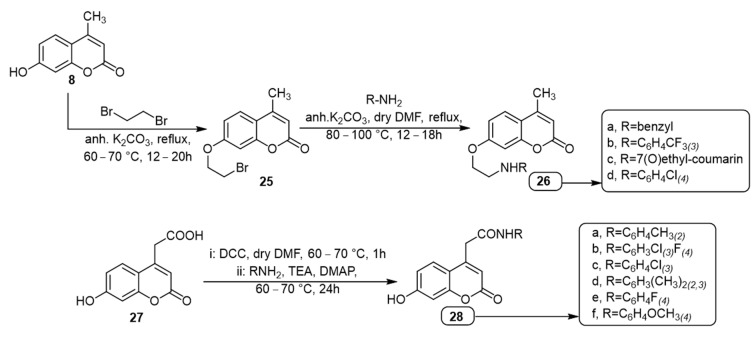
Synthesis of C-7(O) modified 4-methyl coumarinylamines (**26a**–**26d**) and 7-hydroxycoumarinyl acetamides (**28a**–**28f**).

A series of C-7(O) modified 4-methyl coumarinylamines (**26a**–**26d**) and 7-hydroxycoumarinyl acetamides (**28a**–**28f**) have been synthesized according to Scheme 2 and were screened for their antibiofilm activity against *S. aureus* and *P. aeruginosa* [78]. Compounds **26a**–**26d** were obtained via the reaction of compound **8** with 1,2-dibromoethane under reflux in the presence of anhydrous K_2_CO_3_ to yield compound **25** as an intermediate. The reaction of the intermediate **25** with different primary aromatic amines in dry *N*,*N*-dimethylformamide and in the presence of anhydrous K_2_CO_3_ under reflux afforded C-7(O) modified 4-methyl coumarinylamines (**26a**–**26d**) (Scheme 2). Compounds **28a**–**28f** were prepared via heating of 2-(7-hydroxy-2-oxo-2H-chromen-4-yl)acetic acid (27) with dicyclohexyl carbodiimide (DCC) in dry DMF. Then, different aromatic amins were added in the presence of a catalytic amount of triethylamine and 4-dimethylaminopyridine (DMAP) to afford the corresponding 7-hydroxycoumarinyl acetamides (**28a**–**28f**) (Scheme 2). Most of the tested compounds **26a**–**d** and **28a**–**f** showed maximum inhibition of 50% against both *S. aureus* and *P. aeruginosa* [78]. Total protein quantification of surface-adhered biofilm revealed a significant decrease in the biofilm-forming ability of both *S. aureus* and *P. aeruginosa* by **26a** (66.96% and 84.19%) and **26b** (54.73% and 55.06%), respectively. However, compounds **26a** (66.86%), **26b** (66.77%), **28c** (71.91%), and **26c** (71.95%) showed a significant antibiofilm effect against *S. aureus* only. On the other hand, compounds **28d** (60.96%), **28e** (61.56%), **28f** (73.35%), and **26d** (53.97%) showed a maximum antibiofilm effect against *P. aeruginosa* only as revealed by total protein quantification of biofilm [78].

**Scheme 3:**
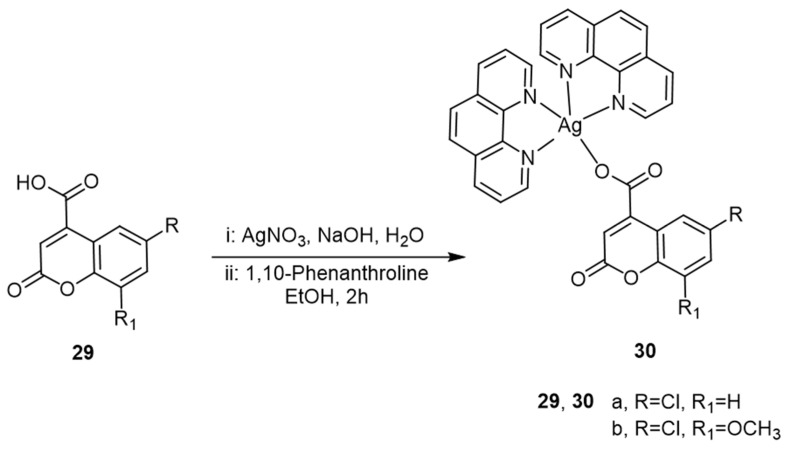
Synthesis of Ag(I) phenanthroline-coumarin complexes **30a** and **30b**.

The Ag(I) phenanthroline–coumarin complexes **30a** and **30b** were synthesized by treating a solution of coumarin acids **29** and silver nitrate with 1,10-phenanthroline in ethanol under stirring for 2 h (Scheme 3). The complexes **30a** and **30b** were screened for their in vitro antibacterial activity against *P. aeruginosa* ATCC 27853. The silver complexes **30a** and **30b** showed minimum inhibition concentration (MIC_90_) values of 55.7 and 48.5 μM against *P. aeruginosa.* These compounds exerted their activity by disrupting the signal processes associated with biofilm formation, thus hindering the mechanisms involved in biofilm resistance to antimicrobial agents [79].

**Scheme 4:**
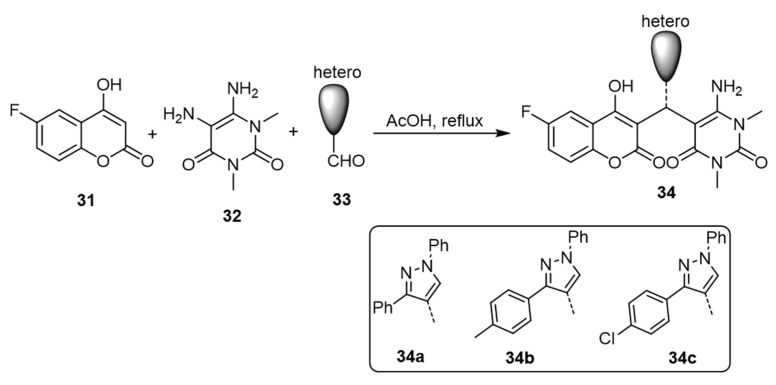
Synthesis of 3-benzylpyrimidino chromen-2-ones (**34**).

A one-pot three-component reaction of 6-fluoro-4-hydroxycoumarin (**31**), 5,6-diamino-1,3-dimethylpyrimidine-2,4(1*H*,3*H*)-dione (**32**), and various heterocyclic aldehydes (**33**) in acetic acid led to the formation of a series of 3-benzylpyrimidino chromen-2-ones (**34**) (Scheme 4). The obtained compounds were evaluated for their antibacterial and antibiofilm formation activities against *Micrococcus luteus* MTCC 2470, *S. aureus* MTCC 96, *S. aureus* MLS-16 MTCC 2940, *Bacillus subtilis* MTCC 121, Gram-positive bacterial strains, and *P. aeruginosa* MTCC 2453, which is a Gram-negative bacterium. Compounds **34b** and **34c** showed anti-*M. luteus* activity with a MIC of 0.55 μg/mL and were equivalent to ciprofloxacin. Additionally, both coumarin derivatives were able to inhibit *M. luteus* biofilm formation at a minimum biofilm eradication concentration (MBEC) of 4 μg/mL. On the other hand, compound **34a** had fair antibacterial activity against *M. luteus* MTCC 2470, *S. aureus* MTCC 96, and *S. aureus* MLS-16 MTCC 2940 with MICs of 2.34, 2.34, and 1.17 μg/mL, respectively. Meanwhile, **34a** inhibited *S. aureus* MTCC 96 biofilm with a MBEC of 4 μg/mL [80]. 

**Scheme 5:**
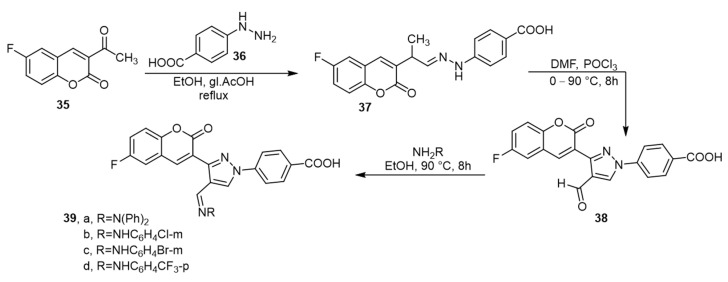
Synthesis of fluoro-coumarin-substituted pyrazole-derived hydrazone derivatives.

Fluoro-coumarin-substituted pyrazole-derived hydrazones (**39a**–**39d**) have been synthesized as outlined in Scheme 5. First, the reaction of fluoro-3-acetyl coumarin (**35**) with 4-hydrazinobenzoic acid (**36**) in ethanol and in the presence of glacial acetic acid gave the hydrazone product (**37**). The latter compound under the Vilsmier-Haack formylation reaction yielded the corresponding pyrazole aldehyde derivative (**38**). The reaction of compound (**38**) with various primary aromatic amines afforded the target fluoro-coumarin-substituted pyrazole-derived hydrazones (**39a**–**39d**) (Scheme 5) [81]. Compounds 39a and **39d** showed notable activity against MRSA, with MICs as low as 3.125 µg/mL. Additionally, **39a** inhibited biofilm formation by 85% of its half MIC, while **39d** removed more than 90% of the biofilm at 2× and 1× of its MICs. Although compound **39b** showed moderate antimicrobial activity with a MIC that did not exceed 12.25 µg/mL, it inhibited biofilm formation at 2× and 1× of its MIC values. These results were very significant, as vancomycin (the positive control) could only eliminate ~70% of the preformed biofilm at 2× of its MIC (0.78–3.125 µg/mL) [81].

**Scheme 6:**
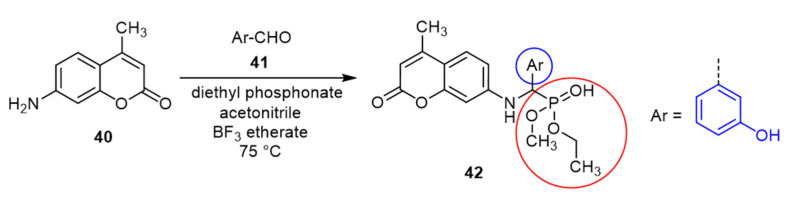
Synthesis of 3-hydroxylphenyl aminophosphonate (**42**).

Yang et al. prepared a series of coumarin aminophosphonates as coumaphos analogs (insecticide reveals great medical potential in agriculture) via the reaction of 4-methyl-7-aminocoumarin (**40**) with various aldehydes (**41**) in the presence of diethyl phosphonate (Scheme 6). The bioactivity assessment identified that 3-hydroxylphenyl aminophosphonate (**42**) exhibited profound in vitro inhibition potency against *S. aureus* at low concentrations (0.5 μg/mL). Also, **42** was capable of eradicating the *S. aureus* biofilm, thus alleviating the development of *S. aureus* resistance. Thus, it was able to destroy the integrity of the cell membrane, which resulted in protein leakage and metabolism inhibition. Biofilm formation decreased significantly by 30% at a concentration of compound **42** that was 16 times its MIC [82].

**Scheme 7:**
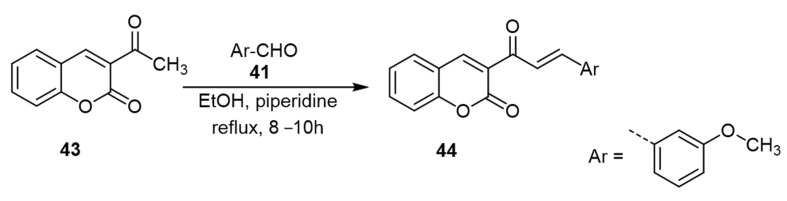
Synthesis of (E)-3-(3-(3-methoxyphenyl) acryloyl)-2H-chromen-2-one (**44**).

Zhang et al. prepared a series of coumarin–chalcone derivatives using the basic catalyzed reaction of 3-acetyl coumarin (**43**) and various aldehydes (**41**) [Claisen–Schmidt condensation reaction] (Scheme 7). Out of these compounds, (E)-3-(3-(3-methoxy-phenyl)acryloyl)-2H-chromen-2-one (**44**) decreased *P. aeruginosa* (PAO1) biofilm biomass by 70%, demonstrating strong biofilm formation inhibition with IC_50_ values ranging from 0.5 and 5 mM without significant impact on the growth of PAO1 or motilities [83]. They suggested that the bioactivity of the methoxy compound depends on reducing the transcription of the exopolysaccharide (PSL) operon to decrease their production, leading to a 60% reduction in its production. Additionally, the methoxy compound reduced the synthesis of c-di-GMP, an important signaling molecule controlling biofilm formation, lowering its concentration in PAO1 by twofold. Furthermore, the methoxy compound reduced the transcription of eight genes, namely, PA4843 (gcbA), PA4396, PA1107 (roeA), PA4929 (nicD), PA3343 (hsbD), PA0290, PA5487 (dgcH), and PA0847, which encode diguanylate cyclases (DGCs) in *P. aeruginosa* to synthesize c-di-GMP. Moreover, the methoxy compound significantly reduced the virulence of *P. aeruginosa* by inhibiting the transcription of several QS regulators, especially interfering with the function of LasR and PqsR, which are two key QS regulators. Hence, they concluded that the methoxy compound inhibits biofilm formation and reduces the virulence of *P. aeruginosa* by repressing c-di-GMP, Psl production, and the QS system.

**Scheme 8:**
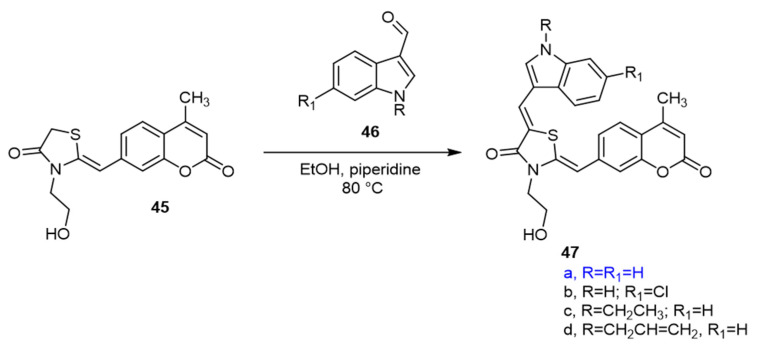
Synthesis of conjugated indole coumarin thiazolidinone compounds (**47a**–**47d**).

Yung et al. prepared unique conjugated indole coumarin thiazolidinone compounds (47a–47d) as novel broad-spectrum antibacterial agents with the basic catalyzed reaction of (Z)-3-(2-hydroxyethyl)-2-((4-methyl-2-oxo-2H-chromen-7-yl)methylene)thiazolidine-4-one (**45**) with various 1,5-disubstituted indole aldehydes (**46**) (Scheme 8). Compound **47a**, with low cytotoxicity to mammalian cells, exhibited a wide antibacterial spectrum and demonstrated potent inhibition efficiencies against Gram-positive and Gram-negative bacteria at low concentrations (0.25–2 μg/mL) compared to clinical norfloxacin. Furthermore, compound **47a** eradicated the burden of *Acinetobacter baumannii* biofilm by 45% at 2 μg/mL (8 × MIC), which could potentially alleviate the development of drug resistance, as it had the ability to damage cell membranes, leading to protein and DNA leakage and hindering normal metabolism [84].

**Scheme 9:**
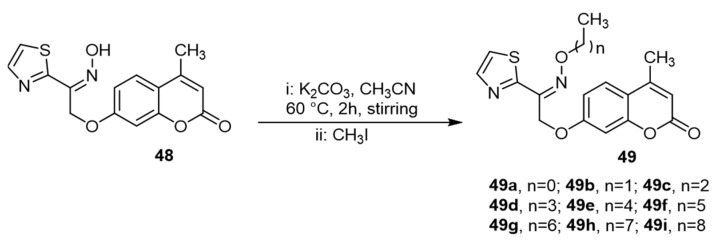
Synthesis of coumarin-containing thiazoles (**49a**–**49i**).

Coumarin containing thiazoles (**49a**–**49i**) has been synthesized starting from hydroxyethyl-connected coumarin thiazole (**48**) as a unique structural skeleton with antimicrobial characteristics (Scheme 9). The compound (Z)-7-(2-(methoxyimino)-2-(thiazol-2-yl) ethoxy)-4-methyl-2*H*-chromen-2-one (**49a**), where n = 0, showed potent inhibitory efficacy against MRSA at a low concentration (1 μg/mL), as well as good capability of eradicating MRSA biofilms. Compound **49a** was able to damage the integrity of the bacterial membrane to trigger leakage of proteins, insert itself into MRSA DNA to block its replication, and induce the generation of reactive oxygen species (ROS) to inhibit bacterial growth [85]. 

**Scheme 10:**
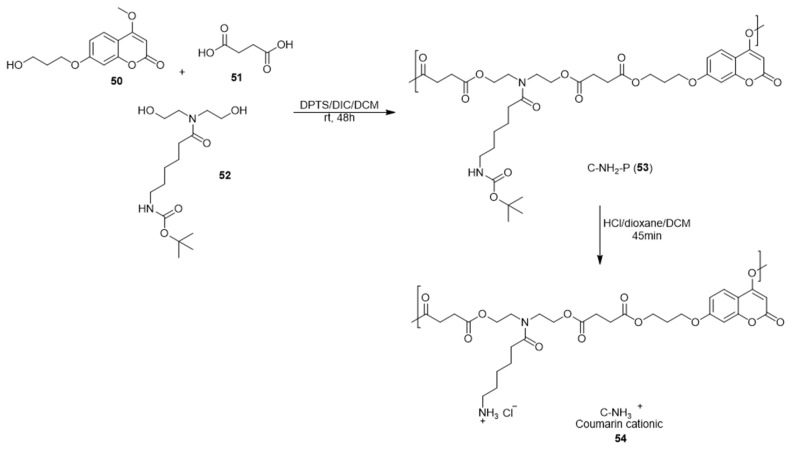
Synthesis of a coumarin polyester with a pendant cationic amine (C-NH_3_ ^+^, **54**).

In the field of polymeric antimicrobial coatings for medical applications, a new coumarin polyester with pendant cationic amine was synthesized (Scheme 10). Coumarin polyester (protected amine) (C-NH_2_-P, 53) was synthesized via the reaction of 7-(3-hydroxypropoxy)-4-methoxy-2H-chromen-2-one (**50**), succinic acid (**51**), and tert-butyl (6-(bis(2-hydroxyethyl) amino)-6-oxohexyl)carbamate (**52**) in the presence of 4-(*N*,*N*-dimethylamino)pyridinium-4-toluenesulfonate (DPTS). The latter reaction mixture was treated with *N*,*N*-diisopropylcarbodiimide (DIC) in dichloromethan to give C-NH_2_-P (**53**) after standing for 48 h at room temperature. Deprotected coumarin polyester (**54**) was obtained via the acid hydrolysis of C-NH_2_-P (**53**) with 4N HCl in dioxane. The new coumarin polyesters were coated onto glass coverslips, and their antimicrobial activity against *P. aeruginosa* colonization on the surface was assessed. The cationic amine polyester (C-NH_3_^+^, **54**) showed a remarkable ability to kill the attached *P. aeruginosa* cells, resulting in the absence of biofilm formation; this effect arises from the pendant cationic charge and is not due to the leaching of small molecules or oligomers from the polymeric matrix [86].

## 5. Synergy between Antibiotics and Coumarin Compounds 

**Figure 6:**
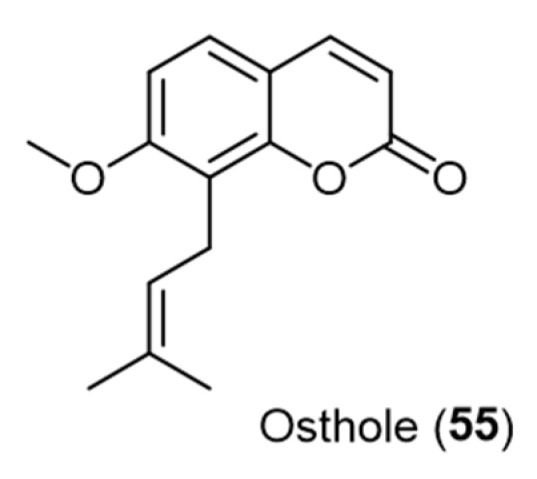
Chemical structure of osthole (**55**).

Different studies showed that coumarins have synergistic effects when combined with other antibiotics and antifungal agents. Osthole (**55**) is a natural coumarin derived from the *Cnidium monnieri* plant that inhibits *C. albicans* SC5314 growth and biofilm formation (Figure 6). Li and his colleagues showed that both osthole (**55**) and fluconazole alone did not exhibit an antibiofilm effect. However, combining fluconazole and osthole (**55**) had a significant synergistic effect by reducing *C. albicans* biofilm formation by 90%. An expression profile microarray revealed that the combined treatment of fluconazole and osthole induced significant changes in the expression of several genes. These genes were associated with oxidation–reduction processes and energy metabolism [87,88,89].

Another study demonstrated that the addition of plant-derived coumarins to the culture of *C. violaceum* ATCC 31532 inhibited its growth. Additionally, combining amikacin with coumarin inhibited QS-dependent violacein biosynthesis. Thus, minimal concentrations of amikacin were required for 50% suppression of violacein biosynthesis in the presence of coumarin. A superadditive anti-QS effect was observed while testing a whole range of subinhibitory concentrations of both amikacin and coumarin. This superadditive anti-QS effect was due to a nonspecific decrease in bacterial cell sensitivity to the autoinducer C_6_-AHL [90]. Additionally, coumarin displayed antimicrobial and antibiofilm effects on *S. typhimurium.* It inhibited extracellular matrix production by suppressing cellulose and curli fimbriae production through downregulating three main biofilm regulatory genes, namely curlin subunit gene D (*csgD*), curlin subunit gene A (*csgA*), and adhesion-related gene A (*adrA*). Coumarin also reduced the flagellar motility of *S. typhimurium*. Another study demonstrated that when coumarin is combined with resveratrol, it exhibited improved antibiofilm properties compared with each compound alone, leading to an additive effect [91]. Similar improved antibiofilm activity against *P. aeruginosa* was observed when coumarin derived from *Angelica dahurica* was combined with ampicillin and ceftazidime [92].

In 2022, a global proteomic profiling of several *P. aeruginosa* strains treated with umbelliferone (**5**) was conducted. The protein–protein interaction network analysis demonstrated that UMB differentially regulates the proteins involved in QS, virulence, stress responses, and the biosynthesis of secondary metabolites. Additionally, gene enrichment analysis showed that UMB treatment regulated several genes responsible for *P. aeruginosa* molecular processes, such as proton-transporting ATP synthase and the succinate–CoA ligase complex. Many major *P. aeruginosa* virulence-associated proteins, including RhlR, LasA, and AlgL, were suppressed upon UMB treatment. Pyocyanin, protease, elastase, and catalase were also reduced, confirming the anti-virulence capacity of UMB against different strains of *P. aeruginosa*. UMB treatment enhanced antibiotic susceptibility to various antibiotics, including amikacin, tobramycin, and cefotaxime [93]. Thus, the synergistic effect resulting from the coumarin–antibiotic combination could be a promising approach for significantly reducing the usage of antibiotics against bacterial resistance.

### Quorum Sensing and Antibiofilm Formation: Theoretical Studies of Coumarin Compounds

A number of theoretical studies addressed the activity of coumarin and its derivatives (natural or synthetic) as biofilm inhibitors in order to identify their mode of action. In 2023, Khalil and his coworkers used computational methods including molecular docking, ADMET properties, and the Lipinski rule of five to study the antimicrobial activity of ten bioactive compounds from *Reynoutria japonica* as quorum quenchers. The molecular docking studies showed the binding modes of the compounds to the active pockets of the target proteins of *S. aureus*: AgrA, AgrB, AgrC, and TRA. Coumarin (**1**) showed a -6.6 Kcal/mol binding score with one hydrogen bond with the amino acid (ASN353) of the active pocket and seven hydrophobic interactions with LEU381, LEU365, ASN323, PHE405, ILE359, ALA327, and CYS355. On the other hand, physiochemical and pharmacokinetic properties indicated that coumarin did not obey the Lipinski rule of five [94]. 

Qu and his colleagues showed that DCH’s anti-MRSA activity likely occurred through targeting bacterial arginine repressor (ArgR). ArgR is the master regulator of *arcABCD* operon expression in many bacterial species, and the presence of arginine results in decreased binding of ArgR to the *arc* promoter, allowing arginine to be catabolized through the *arc*-encoded enzyme degradation pathway. They studied the molecular docking of DCH (**24**) toward the C-terminal domain of *S. aureus* arginine repressor (*S. aureus* ArgR_C_). The molecular docking results indicated that DCH (**24**) binds to arginine in the active pocket of ArgR_C_ and recorded a docking score of −5.07 Kcal/mol. It showed two obvious interactions between the DCH’s benzene rings and GLN25 and ASP46 amino acids in the active pocket of ArgR_C_. Thus, the molecular docking study provided evidence that DCH targets the arginine repressor ArgR of *S. aureus* [77].

In 2021, molecular docking studies were conducted to identify the mode of interaction between 6-methycoumarin (6MC, **21**) and QS receptors of *P. aeruginosa* and *C. violaceum* [69]. The structures of QS activator proteins from *P. aeruginosa*, including LasI (acyl-homoserine-lactone synthase), LasR (transcriptional activator protein receptor protein), PqsE (pseudomonas quinolone signal response protein), and SidA (QS transcription regulator protein), as well as the structure of the QS activator protein from *C. violaceum* CviR, were used. The molecular docking study verified that 6MC showed a higher binding affinity toward Rhl systems than LasI with a docking score of −7.9 kcal mol^−1^ and revealed its interaction with Tyr55 and His-53 (π-π non-covalent interaction) as well as Leu-42 (π–sigma interaction) [69]. In a similar manner, 6MC binds to the active pocket of CviR with a binding energy score of −6.8 kcal mol^−1^ and forms two hydrogen bonds with the active amino acids Gln-90 and Ser-89. Hence, by comparing the binding affinity and types of bond formed, 6MC was demonstrated to be a potential QS inhibitor against *P. aeruginosa* compared to *C. violaceum* [69]. As a result, in both bacteria, 6MC mainly binds to the QS system receptor [69].

In a later study in 2021, Abul Qais and others studied the mode of action of coumarin (**1**) against the active site of acylhomoserine lactone (AHL) synthases and QS regulatory proteins. Coumarin (**1**) was tested against the QS-regulated virulent traits of Gram-negative bacteria, including *C. violaceum ATCC* 12472, *P. aeruginosa* PAO1, and *Pantoea stewartii* [24]. The results showed that coumarin (**1**) bound to the active sites of proteins, including AHL synthases and regulatory proteins (Table 2), forming a stable complex. Table 2 displays the Gram-negative bacterial proteins targeted by coumarin (**1**). 

Ding and colleagues analyzed the effectiveness of daphnetin (**7**), which was shown to reduce the production of extracellular polysaccharides and virulence factors in *R. solanacearum* in a molecular docking study using the EpsB protein [65]. Eps genes are necessary for biofilm formation and virulence in tobacco plants. The molecular docking results indicated that daphnetin showed a favorable binding mode inside the active pocket of EpsB protein. Moreover, daphnetin showed a binding energy score of −4.25 Kcal mol^−1^ and interacted with four key amino acids (LEU222, ARG227, ALA285, and GLU286) in the active pocket of EpsB via conventional hydrogen bonds and hydrophobic interactions. The hydroxyl group at position 8 of daphnetin formed a conventional hydrogen bond with ALA285 in addition to Van der Waals interactions with the acidic residues ALA213, ASP225, SER226, GLU283, GLU284, VAL290, and LEU289 of the active pocket of EpsB. This indicated that daphnetin can be considered a specific ligand for EpsB protein [65]. 

## 6. Conclusions

This review shows how well coumarin and its derivatives (plant-derived and synthetic) function as QS and biofilm formation inhibitors. Coumarin derivatives, especially the hydroxylated forms, represent an interesting group of compounds to be used as anti-virulence agents against a wide range of pathogens. Thus, coumarin and its derivatives should be considered for the treatment of pathogens and biofilm-associated infections. Moreover, the applications of coumarin derivatives can be expanded in the medical field, where they can be used as coating material for medical tubes to prevent the growth and spread of MRSA through catheters. In addition, animal studies and clinical trials are necessary to demonstrate the effects of different coumarin derivatives on various aspects and to confirm the in vitro findings. Collectively, all the above mentioned studies open the door to the identification and development of new coumarin derivatives to act as antimicrobial agents through the inhibition of QS and biofilm formation.

## Figures and Tables

**Table 1:** Quorum sensing activity and biofilm formation inhibition of natural coumarins.

Coumarin	Target Microorganism	Mode of Action	Ref
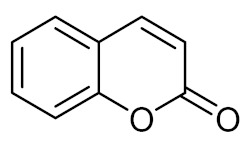 Coumarin (**1**)	*E. coli* O157:H7	Inhibited *E. coli* O157:H7 biofilm formation by >80% without affecting bacterial growth.	[56]
	*S. typhimurium SL1344*	Showed strong antibiofilm effects on *S. typhimurium* at 2.5 μM without showing microbicidal effects. Showed antibiofilm effects against *S. typhimurium SL1344* with an 72% reduction at 2.5 μM and an 99% reduction at 5 μM. Coumarin affects the production of extracellular substances as well as the motility of aggregates necessary for the establishment of mature biofilms, hindering the establishment of robust biofilms.	[57]
	*R. solanacearum*	Showed high bactericidal effects against *R. solanacearum.* Showed no inhibitory effects against *R. solanacearum* biofilm formation after 24 or 48 h of incubation. Showed a slight inhibitory effect on *R. solanacearum* biofilm formation at 72 h, where it damaged bacterial cell membranes and prevented swarming motility and biofilm formation. Significantly inhibited early adhesion and colonization of *R. solanacearum* on tobacco plants by 68% after incubation for 13 days.	[58]
	*C. albicans*	Inhibited fungal adhesion and biofilm formation and destroyed preformed biofilm.	[59]
	*C. violaceum 12472* *P. aeruginosa PAO1* *Serratia marcescens MTCC 97*	Inhibited the production of violacein pigment in *C. violaceum* 12472 by 64.21%. Inhibited the production of pyocyanin, pyoverdin, proteolytic activity, lasB elastase activity, swimming motility, and rhamnolipid production by 87.25, 70.05, 76.07, 58.64, 48.94, and 81.20%, respectively, in *P. aeruginosa* PAO1. Suppressed the virulence factors of *S. marcescens MTCC 97* by more than 50% at the highest subminimum inhibitory concentration.	[24]
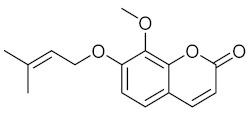 Lacinartin (**14**)	*P. gingivalis*	Inhibited *P. gingivalis* growth. Inhibited *P. gingivalis* biofilm formation by 75% at 50 and 100 μg/mL. Absorbed around one-third of biofilms at concentrations of 12.5–100 µg/mL.	[60]
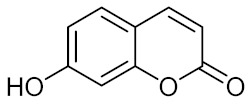 Umbelliferone (UMB, **5**)	*S. aureus* CECT	Inhibited the formation of *S. aureus* CECT 976 biofilm by 50% at 800 μg/mL.	[61]
	*Methicillin-Resistant S. epidermidis (MRSE)*	Showed significant inhibition of biofilm activity by 83% at 500 µg/mL without growth alteration. Reduced the expression of genes related to adhesion (*icaD, atlE, aap, bhp, ebh, sdrG,* and *sdrF*). Downregulated *agrA* and reduced the production of secreted hydrolases upon treatment. Hindered slime synthesis and biofilm matrix components, which were believed to augment antibiotic susceptibility.	[62]
	*P. aeruginosa*	Hindered *P. aeruginosa* biofilm formation on polystyrene and glass surfaces at 125 µg/mL. Downregulated major virulence-associated proteins, such as RhlR, LasA, AlgL, FliD, Tpx, HtpG, KatA, FusA1, Tsf, PhzM, PhzB2, CarB, DctP, MtnA, and MscL. Modulated the global regulators, enzymes, co-factors, and transcription factors related to QS, stress tolerance, siderophore production, motility, and microcolony formation. Showed anti-virulence activity by reducing pyocyanin, protease, elastase, and catalase production in various strains of *P. aeruginosa.* Enhanced the antibiotic susceptibility to various antibiotics including amikacin, kanamycin, tobramycin, ciprofloxacin, and cefotaxime.	[63]
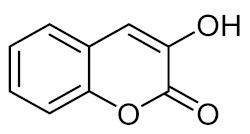 3-Hydroxycoumarin (**2**)	*C. albicans* 90028	Exhibited *C. albicans* antibiofilm activity with a MBIC of 250 µg/mL. Revealed significant downregulation of virulence genes (*hst7*, *ume6*, *efg1*, *cph1*, *ras1*, *als1*) associated with adhesion and morphogenesis.	[64]
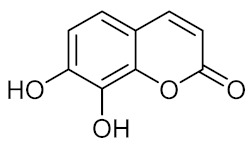 Daphnetin (**7**)	*R. solanacearum*	Significantly reduced extracellular polysaccharides (EPS) production and biofilm formation of *R. solanacearum* through inhibiting the expression of EPS biosynthesis genes (*epsA*, *epsP*, *epsB*, *epsC*, *wecC*, *epsE,* and *epsF*) and the transcriptional regulator factor xpsR, delaying tobacco bacterial wilt disease progression and reducing bacterial density in the stem.	[65,66]
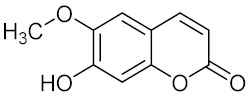 Scopoletin (**12**)	*MDR Candida tropicalis*	Suppressed the formation of elongated fungal forms, the growth rate of established biofilms, and biofilm formation on the surface of coverslips.	[67]
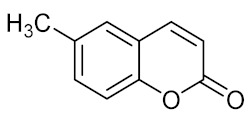 6-Methylcoumarin (**16**)	*R. solanacearum*	Showed potential antibacterial activity. Induced bacterial cell growth into long filaments by suppressing the bacterial division genes *ftsZ* and *ftsQ*.	[68]
	*P. aeruginosa*	Inhibited *P. aeruginosa* PAO1 biofilm formation at 125 μg/mL. Inhibited virulence factors such as pyocyanin, siderophore, exopolysaccharide, elastase, and proteases, which are involved in bacterial motility.	[69]
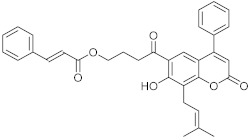 Cinnamoyloxy-mammeisin (CNM, **17**)	*S. aureus* *MRSA S. aureus*	Showed bacteriostatic activity against methicillin-susceptible and -resistant *S. aureus* strains, with a MIC of 11.3 μM. Reduced bacterial adherence to human keratinocytes and disrupted biofilm formation by reducing cell viability and architecture.	[70]
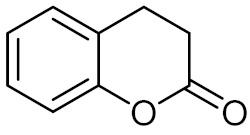 Dihydrocoumarin (**15**)	*H. alvei H4* *C. violaceum* 026	Decreased biofilm formation of *H. alvei H4*, even at subinhibitory concentrations. Inhibited QS via suppression of violacein production in *C. violaceum* 026 and interference with the swimming motility of *H. alvei H4*.	[71]

**Table 2:** Gram-negative bacterial proteins targeted by coumarin (**1**).

Target Gram-Negative Bacteria	Protein	PDB
*P. aeruginosa*	LasI	1RO5
*Pantoea stewartii*	EsaI	1KZF
*P. aeruginosa*	LasR	2UV0
*P. aeruginosa*	LasA	3IT7
*C. violaceum ATCC* 12472	CviR	3QP5
*C. violaceum ATCC* 12472	CviR’	3QP1
*P. aeruginosa*	PqsR	4JVI
*P. aeruginosa*	Rh1R	SWISS-MODEL Repository (ID: P54292)

LasI: acyl-homoserine-lactone (AHL) synthase of *P. aeruginosa* QS; EsaI: acyl-homoserine-lactone (AHL) synthase of *P. stewartii* QS; *LasR protein:* a major transcriptional activator of *P. aeruginosa* QS; Las A: involved in proteolysis and elastolysis (degradation of the host *protein* elastin) of *P. aeruginosa*; *CviR protein*: a key drug target to reduce the virulence properties and pathogenesis of *C. violaceum;* PqsR and Rh1R transcriptional regulators determine the level of the *Pseudomonas* quinolone signal synthesis in *P. aeruginosa.*
